# Self-directed e-learning at a tertiary hospital in Malawi – A qualitative Evaluation and Lessons learnt

**DOI:** 10.3205/zma000949

**Published:** 2015-02-11

**Authors:** Sandra Barteit, Philip Hoepffner, Sören Huwendiek, Angela Karamagi, Charles Munthali, Antje Theurer, Florian Neuhann

**Affiliations:** 1 Heidelberg University, Institute of Public Health, Heidelberg, Germany; 2 University Hospital of Würzburg, Clinic and Polyclinic for Psychiatry and Psychotherapy, Würzburg, Germany; 3 University of Bern, Institute of Medical Education, Department of Assessment and Evaluation, Bern, Switzerland; 4 ESTHER Germany, Malawi German Networking for Capacity Building in Treatment, Training and Research at Kamuzu Central Hospital (MAGNET), Germany; 5 Kamuzu Central Hospital, Medical Department, Lilongwe, Malawi

**Keywords:** computer-assisted instruction, multimedia, Medical Education, capacity, ICT, understaffed, teaching hospital, virtual patients, Sub-saharan Africa

## Abstract

**Background: **Malawi faces a severe lack of health workers. Despite initiatives to address this problem, a critical shortage of health care staff remains. This lack challenges the education and training of junior medical staff, especially medical interns in their final and crucial training year before they independently work as medical doctors.

**Project description: **We have introduced an e-learning platform in the medical department of the Kamuzu Central Hospital (KCH) in Malawi. With the support of computer-assisted instruction, we aimed to improve the quality of medical training and education, as well as access to current medical materials, in particular for interns.

**Method: **From March to April 2012, we conducted a qualitative evaluation to assess relevance and appropriateness of the e-learning platform. Data was collected via face-to-face interviews, a guided group discussion and a checklist based observation log. Evaluation data was recorded and coded using content analysis, interviewees were chosen via purposive sampling.

**Results: **E-learning proved to be technically feasible in this setting. Users considered the e-learning platform to be relevant and appropriate. Concerns were raised about sustainability, accessibility and technical infrastructure, as well as limited involvement and responsibilities of Malawian partners. Interest in e-learning was high, yet, awareness of and knowledge about the e-learning platform among potential users was low. Evaluation results indicated that further adaptions to local needs are necessary to increase usage and accessibility.

**Conclusions:** Interview results and our project experiences showed that, in the given setting, e-learning requires commitment from local stakeholders, adequate technical infrastructure, identification and assignation of responsibilities, as well as specific adaption to local needs.

## Zusammenfassung

**Einführung: **Trotz verschiedener Initiativen mehr Gesundheitspersonal auszubilden, besteht in Malawi weiterhin ein ausgeprägter Mangel an Fachkräften im Gesundheitsbereich. Dieser Mangel stellt auch eine erhebliche Herausforderung für die Aus- und Weiterbildung von medizinischem Personal dar - vor allem für Interns, die in ihrem letzten und entscheidenden Ausbildungsjahr vor dem Beginn einer selbstständigen ärztlichen Tätigkeit stehen.

**Projektbeschreibung: **Wir haben deshalb in der medizinischen Abteilung des Kamuzu Central Hospital (KCH) in Malawi eine E-Learning Plattform eingeführt. Ziel ist es durch den computergestützten Unterricht den Mangel an klinischem Lehrpersonal teilweise zu kompensieren und damit zur Verbesserung der Qualität der medizinischen Ausbildung beizutragen sowie Zugang zu aktuellen und relevanten medizinischen Materialien zu gewährleisten.

**Methoden: **Zwischen März und April 2012 führten wir zur Evaluation von Relevanz und Angemessenheit der E-Learning-Plattform eine qualitative Untersuchung durch. Die Datenerhebung erfolgte über individuelle Interviews, eine thematisch geleitete Gruppendiskussion sowie durch Beobachtung der Nutzung der Plattform basierend auf einer Checkliste. Die Auswahl der Interviewpartner erfolgte gezielt. Interviews und Gruppendiskussion wurden aufgezeichnet, codiert und interpretiert.

**Ergebnisse: **E-Learning erwies sich im Umfeld dieses Krankenhauses als technisch machbar. Die Nutzer beurteilten die E-Learning-Plattform als für sich relevant und inhaltlich angemessen. Bedenken wurden in Bezug auf Nachhaltigkeit, Zugänglichkeit und technischer Infrastruktur geäußert sowie hinsichtlich der begrenzten Beteiligung und Verantwortung der malawischen Partner. Das Interesse an E-Learning war hoch, aber sowohl der Bekanntheitsgrad wie auch die Kenntnisse der Plattform waren unter den potenziellen Nutzern gering. Die Evaluation ergab, dass weitere Anpassungen im Hinblick auf die lokalen Bedürfnisse erforderlich sind, um Nutzung und Zugänglichkeit zu erhöhen.

**Schlussfolgerung:** Um eine aktive Nutzung von E-Learning im gegebenen Umfeld zu erzielen, sind ein aktives Engagement von lokalen Akteuren, eine Identifizierung und Übernahme von spezifischen Verantwortungsbereichen, eine lokal angemessene technische Infrastruktur sowie die Ausrichtung auf die lokalen Bedürfnisse erforderlich.

## Introduction

Malawi faces an immense shortage of health personnel paired with poor access to health information. In 2013, Malawi had a ratio of 0.2 physicians and 3.4 nurses and midwives per 10.000 population [http://www.who.int/countries/mwi/en/], compared to the WHO recommendation of 23 doctors, nurses and midwives [http://www.who.int/hrh/workforce_mdgs/en/]. Initiatives to address this issue included a short 3-year diploma for clinical officers to alleviate the lack of medical doctors [1], extending medical education within Malawi by opening the College of Medicine in 1991 [2], [http://www.medcol.mw/?page_id=1194] and an emergency training plan for health care workers in 2005 [http://www.who.int/countryfocus/cooperation_strategy/ccs_mwi_en.pdf]. Facing the dire lack of senior clinical doctors for supervision and teaching during internship, we introduced an e-learning platform [http://www.esther-magnet.org] in the medical department of the Kamuzu Central Hospital (KCH). The intention was to compensate for the lack of medical teaching staff, to increase access and to improve quality of medical education in the department with the support of information and communications technology (ICT) and by providing access to up-to-date medical information [3]. E-learning programs were successfully used to train healthcare workers in low- and middle-income-countries, like e-learning for evidence-based medicine [4] and for nursing education in Malaysia [5]. We targeted medical interns during the critical phase from trainee to an independent medical professional.

The introduction of e-learning was embedded in a wider approach to improve quality of care and in service training at the medical department, supported by a hospital partnership [http://www.esther.eu].

In the following, we present the concept and implementation of e-learning, as well as its evaluation and corresponding results one year after its introduction.

## Project Description

KCH is a tertiary 800-bed-hospital administered by the Ministry of Health [6]. It serves as a teaching hospital and as a referral hospital for central Malawi with a catchment population of around 6 million [7], [http://www.who.int/patientsafety/implementation/apps/first_wave/malawi_middlesbrough/en/index.html]. The medical department has a bed capacity of around 90 and runs daily outpatient’s clinics, an admitting ward and is further responsible for the tuberculosis ward off campus.

In Malawi, the College of Medicine (CoM) provides full medical training since 1991 [2], [http://www.medcol.mw/?page_id=415]. Currently, there are about forty new medical doctors graduating per year [8], whereby first-year intake numbers are gradually increased [2]. Graduates intern at a hospital, rotating through departments of Medicine, Surgery, Paediatrics and Obstetrics-Gynaecology. After the internship, the majority works in the Malawian public sector [2].

Another essential medical cadre are clinical officers (CO) [1]. They provide medical care, give anesthesia, do surgical procedures and perform around 80% of all Malawian caesarian sections [9]. After 3 years, CO students graduate with a diploma in clinical medicine, followed by a one year hospital internship [1].

The lack of senior medical doctors impairs the learning opportunities for interns, especially during their vital internship-year. During the project period there were two departmental senior doctors responsible for intern training, in addition to their tasks in patient care and administration. Improving the education during the internship is pivotal in order to sustain good quality of treatment and care.

### Implementation and content

The e-learning platform was initially setup by the German project partners with medical materials created within the project. 

#### Technical setup

With a preference for open source software, the e-learning platform was setup in Moodle [http://docs.moodle.org/26/en/About_Moodle], which is also used at the partnering University. The on-site computer equipment consists out of 2 workstations and 2 laptops. To save cost and time, we rented an already setup Linux virtual server, located in Germany. We contracted the Malawi based Internet service provider Globe Internet Ltd. For the Local Area Network (LAN), Globe Internet Ltd. installed a WiMAX antenna. The maximum data transfer rate was 256kbps, full duplex. Currently, the department uses a prepaid 3G mobile network with a data transfer rate of up to 21Mbps.

##### Content

The needs of medical interns guided the content creation for and the design of the e-learning platform, particularly considering local relevance and country-specific pathology [2, 6, 7]. Educational needs were specified according to the requirements in the interns’ logbook of the department that has been developed with senior medical staff and project partners. Content is geared to support rotating interns to meet the defined requirements at the end of their rotation with regards to practical knowledge for patient management and demonstration of skills - e.g. lumbar puncture. 

**Figure 1:**
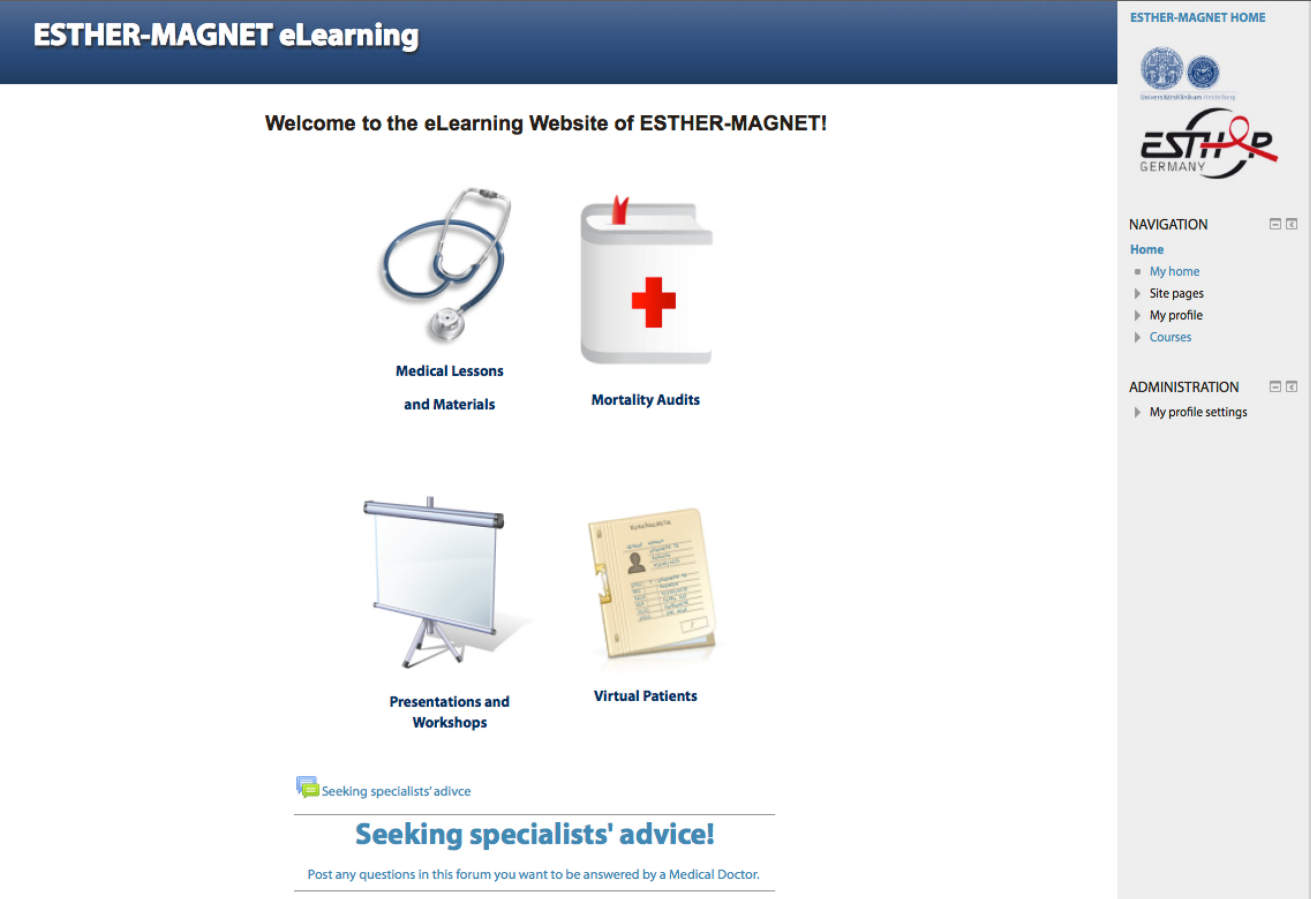
Homepage of the e-learning platform with the four sections: Medical lessons and materials, mortality audits, presentations and workshops, virtual patients.

The platform is organized into four sections: medical lessons and materials, mortality audits, presentations and workshops, and virtual patients (see Figure 1 (Fig. 1)). Virtual patients (VP) are a focal element of the e-learning platform and defined as “an interactive computer simulation of real-life clinical scenarios for the purpose of healthcare and medical training, education or assessment” [10], [11] (see Table 1 (Tab. 1) and 2 (Tab. 2)). Other projects and studies prove the relevance of VPs for healthcare education and their successful application in developing countries [12]. Also, the platform features the medical online libraries HINARI [http://www.who.int/hinari/en/] and UpToDate [http://www.uptodate.com]. Access to the platform is restricted to clinical personnel and interns.

##### Didactics

Interns learn self-directed: they work through medical texts, watch recorded presentations and step-by-step work through VP, which offer also direct feedback to each decision made, multiple-choice questions and references to further relevant materials on third-party websites. Knowledge acquisition during these self-directed learning phases is supported by multimedia activities that cover information, communication and exercise activities to rapidly qualify learners with instructional knowledge [13], [14]. 

## Evaluation

We qualitatively evaluated the project in May 2012, one year after its introduction, focusing on the research question what factors influence the use of the e-learning platform.

### Methods

The data was obtained via recorded face-to-face interviews following a semi-structured interview guide, an interview guided group discussion and a checklist based observation for ICT proficiency. The evaluation was designed after the Quality Standards for Development Evaluation from the Organization for Economic Co-operation and Development (OECD) [http://www.oecd.org/daf/competition/prosecutionandlawenforcement/47381304.pdf], focusing solely on relevance and appropriateness. Interviews were structured into four sections: personal information, access to computers/ICT, general learning and e-learning experiences, and use of e-learning platform. Twenty (3 administrative managers, 2 senior doctors, 2 clinical officers, 9 intern medical doctors and 4 intern clinical officers) interviewees were purposively sampled. 8 interns were invited for group discussion and 6 (3 intern medical doctors, 3 intern clinical officers) randomly selected interns participated in the observation log. Interviews were transcribed and coded using content analysis. The data was analyzed with a word processing program.

#### Results

##### Sustainability

**Figure 2:**
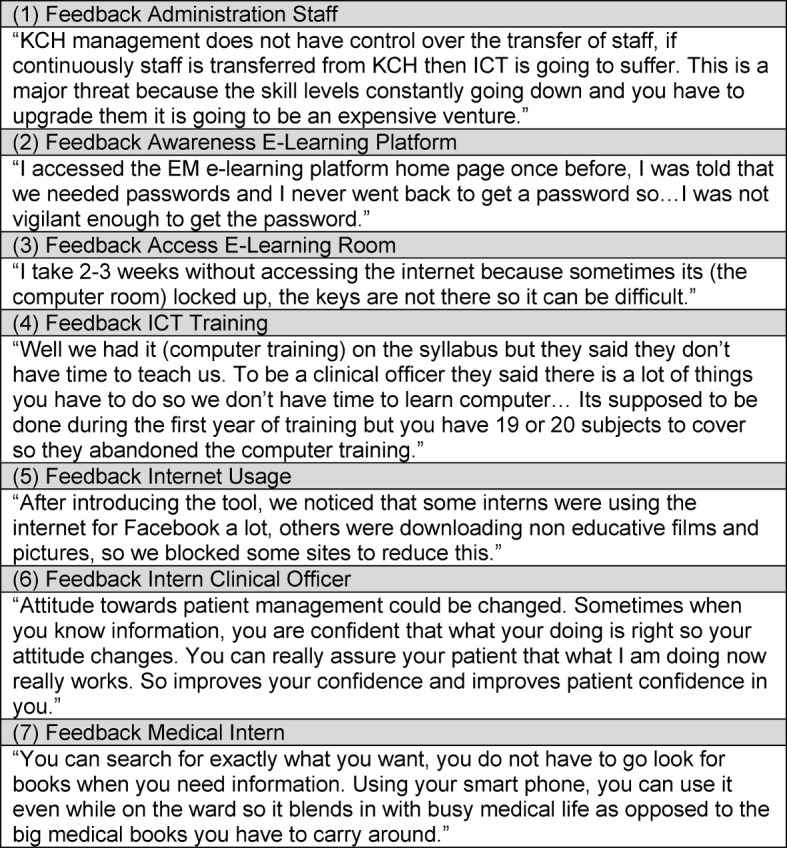
Feedback interviewees.

Concerns were raised about sustainability of the e-learning platform, due to staff shortages and limited integration of local stakeholders. Uncertainty was mentioned with regards to staff retention since KCH is not in charge of staff management and relocation (see Figure 2 (Fig. 2), (1)). Interviewees perceived that the Malawian partners had limited involvement because no local manager for the platform was trained and in charge for administration or support. Therefore, sustainability was rated low and perceived as a major challenge.

##### Awareness and Access

Interns stated that they were not aware of the e-learning platform and that they were told informally about the platform in handover meetings or on ward rounds (see Figure 2 (Fig. 2), (2)). None of the interviewees had a formal introduction to the platform. Some users were impeded using the e-learning platform since the keys to access the computer room stayed with one person, who was not always around (see Figure 2 (Fig. 2), (3)).

##### ICT proficiency

The level of ICT-proficiency varied between intern medical doctors and intern clinical officers. While intern medical doctors are trained in ICT usage during their studies, intern clinical officers reported to receive either no or little ICT training (see Figure 2 (Fig. 2), (4)). One intern clinical officer needed support with basic computer tasks, another intern medical doctors, as well as 2 intern clinical officers showed proficiency for all checkpoints on the observation checklist. Interviewees stated that if they felt the content was of interest to them, they feel encouraged to use ICTs. Several intern clinical officers expressed a need for training in the usage of the e-learning platform and general ICT usage.

##### ICT infrastructure

Although 1 out of 4 intern clinical officers and 5 out of 12 intern medical doctors had access to a private laptop, interns perceived the numbers of computers as insufficient to cover the departmental ICT need, as there are peak-times for computer usage in the medical department. Usage is high during lunchtime and after the workday. Non-professional Internet usage was remarked as a negative side effect (see Figure 2 (Fig. 2), (5)).

##### Relevance

Interviewees confirmed the relevance of content and appropriateness of the e-learning platform to their setting. The medical staff identified the e-learning platform as beneficial to compensate for the lack of senior medical staff and for limitations in access to current and relevant medical information.

##### Potential effects

Respondents viewed the e-learning platform as positive and beneficial. It was affirmed that the e-learning platform improves their clinical skill set, especially in regard to patient management (see Figure 2 (Fig. 2), (6)). Furthermore, the e-learning platform was perceived as a central resource of reliable and up-to-date medical information that reduces effort and time for research (see Figure 2 (Fig. 2), (7)). 

## Discussion

We described the development, technical set up, implementation and user experiences of the e-learning platform as part of a broader approach to improve quality of training and care at the medical department of the KCH.

Various e-learning approaches in developing countries have shown the appropriateness, relevance and the great interest of users [5], [8], [15] to use such a medium for medical training. At KCH, interviewees confirmed these aspects, highlighting the impact on their medical skill set. However, to yield the potential benefits, several issues appear noteworthy: awareness and acceptance, technical challenges and adequacy of IT infrastructure, as well as content design, managerial competence and the need of at least one local e-learning coordinator.

Our evaluation showed that just to provide an e-learning platform is not sufficient. Hence before helping to compensate the lack of clinical teachers, it needs efforts to set up and to maintain it. Medical teachers and involved local staff need commitment to the project [5] to promote awareness, which was perceived low. In our setting, awareness was further impeded by the forced move and temporary shutdown of the computer lab due to reconstruction work of the department for a few months and local financial mismanagement of project funds that cut off Internet access for a longer time period.

Also needed are ICT expertise, infrastructure and support systems, continuous IT support, the commitment and strategies to cooperate, as well as a suitable didactic approach [16]. ICT proficiency to use the platform proved just sufficient, however structured introductions seem advisable for optimal use, as shown in similar e-learning settings [3], [15]. The target group confirmed the positive potential and local preparedness of the technology.

Currently, the number of computers is not sufficient, which has been shown to lower usage [17]. Another infrastructural challenge is the poor broadband network connection with low download speeds diminishing the overall e-learning experience [8]. 

Further, results showed the need for support and dedication of the department, the identification and integration of stakeholders [3] as well as faculty and intern engagement [5].

The critical remarks on lack of involvement and ownership of local capacity have been taken up and we have identified an e-learning coordinator who handles administrative platform tasks, manages content, and offers ICT capacity building and supports interns. As the project started, a person with the respective skills and sufficient time for this task was not available. 

In order to improve awareness, we see potentials in structured introductory sessions for new interns of the department and active promotion of the e-learning platform within the department. Also, the e-learning platform is integrated in the interns’ curriculum. This needs to be an ongoing activity because of the relatively fast turn around of interns who are in the medical ward for 10 weeks.

Rewards have proven to encourage usage and involvement [15]. In the next phase, we take up this approach with a prize competition for interns, so they engage in creating new VP from patient cases or other relevant material for the e-learning platform [15]. 

In order to cut access times and stabilize the network connection, we will test and integrate the following: a general caching system [3] and DNS caching [16], a delay-tolerant network [17], a transfer protocol like BitTorrent [3], off-peak hour downloading for large files and data compression [18]. The range of wireless LAN was extended since interviewees reported to own a laptop.

After the first comprehensive training sessions, we will evaluate didactics and contents of the platform.

Education is a major driving force of a country’s economy [19] and essential to alleviate Malawi’s poverty [20]. ICTs are part of the millennium development goals [http://www.un.org/millenniumgoals] due to their substantial impact on a country’s development [15] and are integrated more and more into the political agenda, such as the Malawian eHealth agenda [17]. As technological access and competition increases, cost for its usage decrease. It makes e-learning a tool of growing importance to sustain and extend access to education, especially in countries that lack skilled health personnel. Studies showed that e-learning has a positive effect in training healthcare workers in similar settings [5], as well as influencing the quality of the training and quantity of trained medical personnel [21].

## Conclusions

We have introduced an e-learning platform at a tertiary hospital in Malawi that centralizes access to relevant and up-to-date medical materials and information that may otherwise be unavailable. It provides a source to refer to standard in treatment guidelines, reduces time for researching and aids the training of medical skills and patient management.

After a qualitative evaluation, we found that for an e-learning platform to work, it requires an appropriate ICT infrastructure with corresponding support, integration of stakeholders and the commitment of the department and involved faculty. Users value the opportunity of an e-learning platform to partially compensate for the lack of clinical teachers, as well as to improve the quality of training for interns and access to medical up-to-date information in the medical department of the KCH.

## Limitations

We appreciate the limitations of our evaluation as we explored only the perceptions of the personnel of one hospital over a limited time span and relatively early after the introduction which limits the potential experience of users. This early evaluation however was done to guide the future development and direction of the platform development. Furthermore, we think the lessons learnt can be helpful for others initiating similar projects.

## Authors’ contributions

All authors were involved in the conception and design of the study and have all approved the final version of the manuscript to be published. All authors have been responsible for redrafting and revising the intellectual content of this article. The corresponding author wrote the first draft, the first and last author contributed equally to the paper.

## Ethical Considerations

Prior to the study, the study protocol was submitted to and approved by the Ethics Committee of the Medical Faculty in Heidelberg (Germany) and the National Health Science Research Committee of Malawi. The management of the KCH approved and permitted the study. Informed consent was given from all respondents prior to interviews and group discussions. Permission to record was also requested. All information shared was kept confidential. The information would not be used to identify them as the respondent.

## Acknowledgements

The hospital partnership project received funding through the German technical cooperation (GIZ), PROFILE (German ESTHER secretariat), within the ESTHER (Ensemble pour une Solidarité Thérapeutique Hospitalière En Réseau) initiative in cooperation with the partnership project MAGNET (Malawi German Networking for Capacity Building in Treatment, Training and Research at Kamuzu Central Hospital).

## Competing interests

The authors declare that they have no competing interests. The authors alone are responsible for the content and the writing of the paper.

## Figures and Tables

**Table 1:**
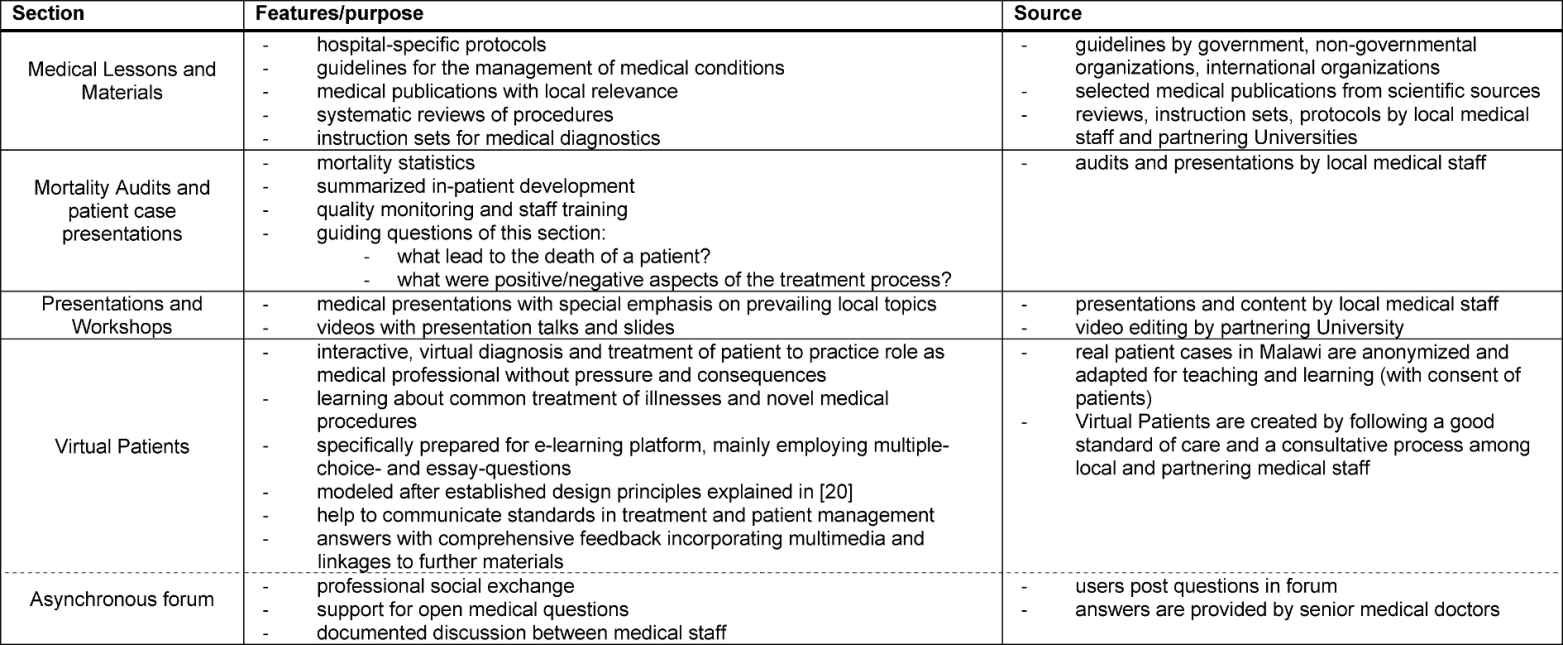
Overview of sections of e-learning platform.

**Table 2:**
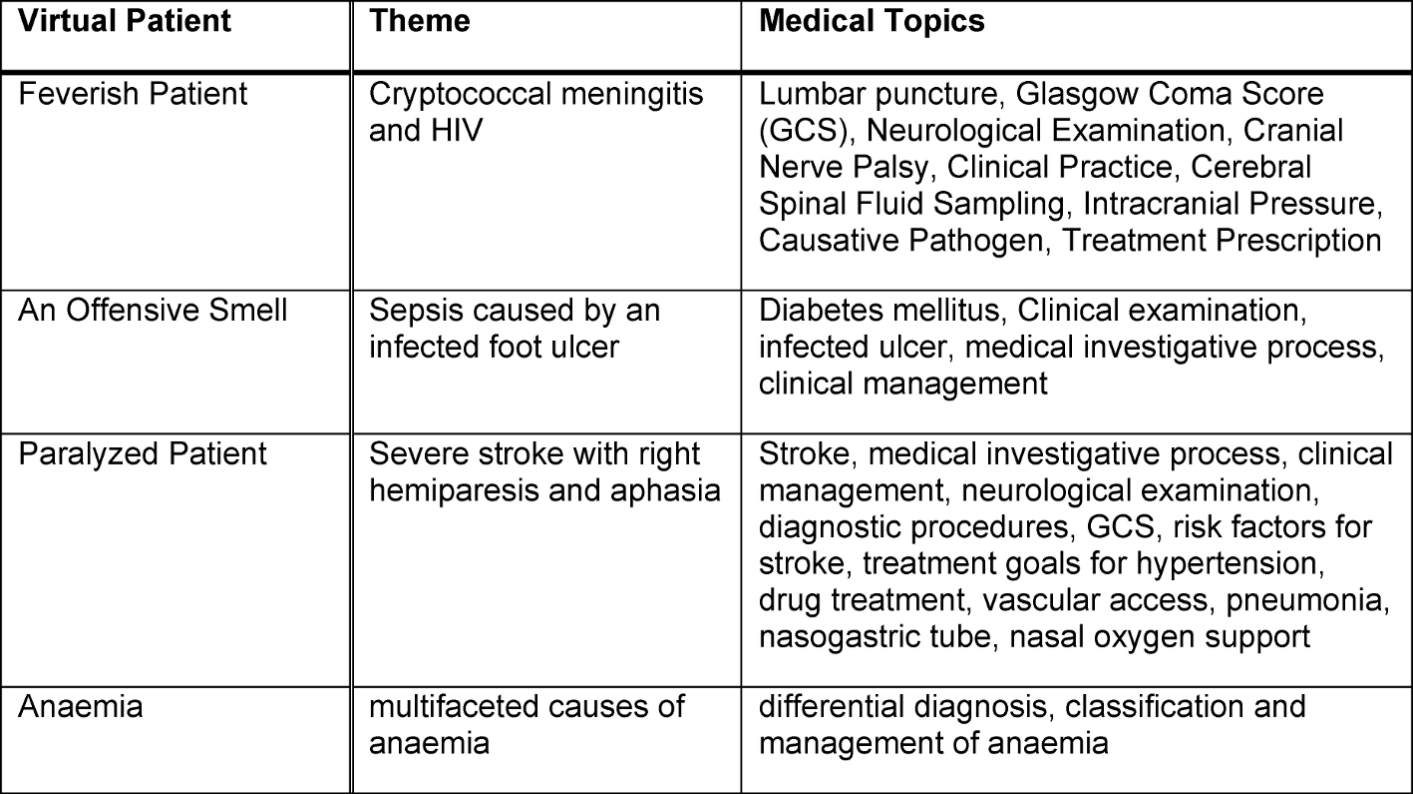
Theme and medical topics of Virtual Patients on the e-learning platform
